# The Course of Study for the "Conjoint"

**Published:** 1901-09-07

**Authors:** 


### The Course of Study for the "Conjoint."

There are certain differences between the course of study required for the various qualifications, but as all the qualifying bodies must conform to the regulations laid down by the General Medical Council, registration by whom alone gives the right to practise, the subjects of study are broadly similar in all cases, the variations being rather in the standard of the examinations and in the time required. Whatever qualification the student seeks he must pass five years in study after he has been registered as a medical student, and before he is so registered he must pass a Preliminary Examination in Arts recognised by the General Medical Council. The list of the examinations which are recognised can be obtained from the Registrar of the General Medical Council, 299 Oxford Street, London, W. It may be well to mention that the matriculation examinations at some of the universities, as for example that of the University of London, is itself recognised as a Preliminary in Arts, so that when it has been passed no other Preliminary is required. Lately there has been a little embroglio between the General Medical Council and the London colleges, the latter of which do not now require that a student must have been registered by the General Medical Council before being admitted to their Examinations. But nevertheless he must pass a Preliminary recognised by the Council, and he must go through his five years of medical study after passing it. Having then become a medical student he will find that the course of study required for the "conjoint" is broadly divided into three very distinct periods, each devoted to a certain class of work and each ending with an Examination by which this work is tested.

The first period is that of the elementary sciences, chemistry, physics, elementary biology and practical pharmacy, and it occupies a year. At certain boys' schools, which are specially recognised for the purpose, these subjects may be studied for a year, after registration as a medical student but before entering a medical school, and the First Examination even may be passed before joining a medical school; or these subjects may be studied before registration as a medical student, but in no case can less than five years elapse between such registration and the passing of the Final Examination. All that these arrangements do is in the first case to shorten the time spent at a medical school and thus to save expense, and in the other case to lighten the work which it is necessary to cram into the five years—sometimes no small consideration.

When the Examination on the above subjects, that is the so-called First Examination, has been passed, the next period is entered into, namely that of the study of anatomy and physiology. This takes fully a year and often more, and then the Second Examination has to be passed, an examination in which a large number of students fail. Having passed this, however, the student at last finds himself in the hospital wards, and the rest of his time is given up to the study of disease and its treatment. Customs differ at different schools, but generally the student is discouraged from spending much time in the hospital until he has passed his Second Examination, and although in the midst of the novelty of the thing the freshman is apt to rush to the wards and the operating theatre, he soon finds out that if he is to pass his Examinations as they become due, his time is pretty well cut out for him, so that he has but little chance of doing anything outside the strict limits of the curriculum.

If he has worked well and passed his Second Examination punctually he will thus find himself full of information as to the structure of the body and with from two and a half to three years in which to study its diseases and their treatment. And it will take him all his time to do it. It is very important to remember that if the Examinations are not passed at the appointed dates the length, and therefore the expense, of the curriculum is increased. The Third or Final Examination may be passed at the end of five years from registration as a medical student. But the proper intervals must be kept. The Final must be four years from the First, and two years from the Second, so that a delay in either of these may push the date of the Final so far on that the student's purse may be exhausted before the time arrives and his labour may be thrown away. Hence the immense importance of every student making a most careful study of the rules and regulations and following the precise course laid down. The discursive enthusiast who wanders into other fields of inquiry, however interesting to him at the moment, is apt to find himself most uncomfortably stranded at the end.

